# Maternal obesity and its impact on pregnancy

**DOI:** 10.6026/973206300214648

**Published:** 2025-12-15

**Authors:** Shambhavi Soni, Himanshy Rai, Mohita Pandey, Abhishek Mehta

**Affiliations:** 1 Department of Obstetrics and Gynecology, Government Medical College, Datia, Madhya Pradesh, India; 2 Department of Obstetrics and Gynecology, Shyam Shah Medical College, Rewa, Madhya Pradesh, India; 3 Department of Microbiology, Government Medical College, Datia, Madhya Pradesh, India

**Keywords:** Maternal obesity, pregnancy complications, cesarean section, postpartum outcomes, body mass index (BMI)

## Abstract

Maternal obesity is associated with a significantly increased risk of complications across all stages of pregnancy. Implementing targeted interventions can help mitigate obesity-related risks and improve overall pregnancy outcomes. Hence, a cross-sectional study was conducted from September 2022 to September 2023 at a tertiary care hospital of Central India to assess the prevalence of obesity among pregnant women and to evaluate its impact on pregnancy complications, mode of delivery and fetal outcomes. Data of 80 pregnant women (BMI >30 kg/m^2^) with obesity, on demographics, medical history, physical activity and dietary practices were collected. Relevant investigations, including glucose tolerance tests, ultrasound scans, blood tests, were performed and Maternal and fetal outcomes were analyzed. Obesity was linked to higher rates of gestational diabetes, hypertensive disorders, and anemia. Intrapartum challenges, including prolonged and preterm labor, were more common, with higher cesarean delivery rates. Postpartum morbidity, particularly hemorrhage, wound infections, and episiotomy dehiscence, was notably higher. These findings highlight the need for integrated strategies to mitigate the adverse effects of obesity on pregnancy.

## Background:

Obesity is one of the most complex and challenging chronic health conditions to manage, with significant implications for maternal and fetal health. The coexistence of obesity and pregnancy introduces additional clinical risks, increasing the likelihood of adverse outcomes for both the mother and the newborn. Rising obesity rates among women of reproductive age have reached epidemic proportions globally, contributing to a substantial burden of antenatal, intrapartum, postpartum and neonatal complications [1]. Multiple factors underline the increasing prevalence of obesity during pregnancy, including poor nutritional awareness, sedentary lifestyles, sociocultural and economic determinants and limited engagement in physical activity. Additionally, obesity is frequently accompanied by pre-existing or pregnancy-induced morbidities, further compounding the risk profile and classifying such pregnancies as high-risk [2]. According to the World Obesity Atlas 2022, over one billion individuals worldwide are living with obesity, including 650 million adults, 340 million adolescents and 39 million children. Projections suggest that by 2025, approximately 167 million people may face health problems attributable to obesity, prompting the World Health Organization (WHO) to call for urgent global action to address this preventable crisis [3]. In the Indian context, the National Family Health Survey (NFHS-5) reported that 24% of women are overweight or affected by obesity- an increase from 21% in NFHS-4 (2015-16). Concurrently, 57.9% of pregnant women remain anemic, compounding the burden of poor maternal health outcomes [4, 5]. Body Mass Index (BMI) remains the most commonly used tool to classify overweight and obesity, with a BMI ≥30 kg/m^2^ defining obesity [6]. Maternal obesity has been associated with a wide range of complications, including miscarriage, gestational diabetes mellitus (GDM), preeclampsia, venous thromboembolism, increased rates of labor induction and cesarean delivery, as well as neonatal outcomes such as stillbirth, macrosomia and congenital anomalies [7, 8]. Therefore, it is of interest to determine the prevalence of obesity among pregnant women and its association with antepartum, intrapartum and postpartum complications, as well as mode of delivery and contribute to the evidence guiding clinical management and preventive strategies.

## Materials and Methods:

## Study design and setting:

A cross-sectional study was conducted from September 2022 to September 2023 in the Department of Obstetrics and Gynecology at G.R. Medical College and J.A. Group of Hospitals, Gwalior, India after seeking approval from institutional ethics committee and obtaining written informed consent from the study subjects.

## Sample size calculation:

Using the NFHS-5 prevalence of obesity among women (24%), a 95% confidence interval and a 10% margin of error, the required sample size was calculated to be 69. Allowing a 15% non-response rate, 80 participants were recruited.

## Participants:

Pregnant women presenting for antenatal care either in the outpatient (OPD) or inpatient (IPD) department were screened.

## Inclusion criteria:

Pre-pregnancy or first-trimester BMI > 30 kg/m^2^.

## Exclusion criteria:

BMI ≤ 29.9 kg/m^2^, multiple gestation, known congenital fetal anomalies, chronic systemic illnesses (e.g., diabetes mellitus, hypertension), intrauterine fetal death or miscarriage before 28 weeks, or refusal to consent.

## Data collection:

At the first antenatal visit, height and weight were measured to calculate BMI. Pre-pregnancy weight was obtained from Mother and Child Protection (MCP) cards or prior OPD records. A structured questionnaire captured demographic data, obstetric history, socioeconomic status (modified Kuppuswamy scale), physical activity levels and dietary modifications. Participants were followed through delivery.

## Clinical and laboratory assessments:

[1] Gestational diabetes mellitus (GDM): Diagnosed by a 75 g oral glucose tolerance test (OGTT) per International Association of Diabetes and Pregnancy Study Groups (IADPSG) criteria.

[2] Gestational hypertension (GHTN), preeclampsia (PE) and eclampsia: Defined according to the American College of Obstetricians and Gynecologists (ACOG) guidelines.

[3] Other investigations: Complete blood count, liver and renal function tests and obstetric ultrasound scans were performed as clinically indicated.

## Outcome measures:

Primary outcomes included the incidence of antepartum complications (GDM, PE, anemia), intrapartum complications (hypertension, prolonged or preterm labour, failed induction), mode of delivery and postpartum complications (atonic/traumatic hemorrhage, wound infection, episiotomy gaping).

## Statistical analysis:

Statistical analysis was performed using SPSS version 20.0, with data presented as frequency distributions, using Tests of significance like Chi-square test, with level of significance set at p < 0.05.

## Results and Discussion:

Eighty pregnant women with obesity (BMI > 30 kg/m^2^) were enrolled, with the greatest prevalence observed in the 18-25 years age group (37.5%), followed closely by those aged 26-30 years (35%). Chi-square test confirmed a significant association between younger age and obesity in this cohort (χ^2^ = 10.45, p = 0.0012). Socioeconomically, 30% of participants were classified as lower class and 35% as middle class. Primiparous women constituted 37.5% of the sample. Pre-pregnancy weights ranged from 61 kg to 90 kg, with nearly half (47.5%) weighing between 71 kg and 80 kg. The mean BMI at first antenatal visit was 32.04 ± 1.74 kg/m^2^. Most women (83.8%) reported no regular physical activity during pregnancy and family history of obesity, diabetes mellitus, hypertension, thyroid disorders and polycystic ovary syndrome were noted in 40.4%, 23.4%, 17.1%, 19.1% and 15%, respectively. In the antepartum period, rates of gestational diabetes mellitus, gestational hypertension and anemia did not differ significantly between women with BMI 30-32 kg/m^2^ and those with BMI > 32 kg/m^2^ (all p > 0.05), whereas severe preeclampsia showed a trend toward higher prevalence in the BMI > 32 kg/m^2^ group (20.8% vs. 7.1%, p = 0.054) (Table 1 - see PDF). During labor, hypertensive complications were significantly more frequent among women with BMI > 32 kg/m^2^ (33.3% vs. 10.7%; p = 0.016), while rates of placental abruption, prolonged labor and preterm labor were similar across BMI groups (all p > 0.05) (Table 2 - see PDF). Postpartum, women with BMI > 32 kg/m^2^ experienced significantly higher rates of wound infection (29.2% vs. 8.9%; p = 0.043) and episiotomy dehiscence (25.0% vs. 7.1%; p = 0.023), whereas incidences of atonic and traumatic hemorrhage did not differ significantly (Table 3 - see PDF). Although cesarean delivery was more common in the higher-BMI group (50.0% vs. 39.3%), differences in primary and repeat cesarean rates, as well as vaginal and vacuum-assisted deliveries, were not statistically significant (all p > 0.05) (Table 4 - see PDF). Overall, elevated BMI was significantly associated with increased intrapartum hypertension and postpartum wound complications, with trends toward greater severe preeclampsia and cesarean delivery warranting further study.

The global prevalence of obesity is rising, often beginning in childhood; for example, 6-7% of Saudi children aged 1-18 years are classified as obese [9]. In our cohort, 80 pregnant women with obesity exhibited a mean body mass index (BMI) of 32.04 ± 1.74 kg/m^2^, reflecting a substantial burden on the population. Higher socioeconomic status was directly associated with obesity, a trend observed in other low- and middle-income settings [10, 11]. Moreover, multiparity was found to increase the odds of obesity by 1.74-fold in comparison to women with fewer births [12]. Endocrine comorbidities were prevalent; small increases in thyroid-stimulating hormone (TSH) were correlated with a 1.9 kg/m^2^ higher BMI [13] and the prevalence of polycystic ovary syndrome (PCOS) was reported at 15%, consistent with previous findings of a bidirectional relationship between obesity and PCOS [14]. Looking at the Antepartum outcomes, obesity significantly heightened the risk of antenatal complications. More than one-third of our subjects developed gestational diabetes mellitus (15%), with a similar proportion experiencing gestational hypertension (16.25%), rates that are comparable to those reported in an Indian cohort, which were 32% and 38%, respectively [15]. Severe preeclampsia was observed in 20% of women with a BMI greater than 32 kg/m^2^, compared to 7.1% in those with lower BMI, yielding a p-value of 0.054 and an odds ratio (OR) of approximately 3 per 10 kg/m^2^ increase in BMI [16]. The incidence of preterm labor was recorded at 17.5%, with a modest increase only at extreme BMI levels (OR 1.26-1.58) [17]. Most of the mothers with obesity were anemic aligning with hepcidin-mediated iron deficiency [18]. In some studies anemia [19] and obesity during pregnancy [20] are in turn associated with subclinical hypothyroidism. While looking at the Intrapartum outcomes, obesity was associated with a twofold increase in labor induction rates and a 2-3-fold increase in the risk of macrosomia (birth weight > 4 kg), corresponding to adjusted ORs of 1.93 and 1.96 in overweight and obese women [13]. Cesarean section rates were recorded at 50% for women with a BMI greater than 32 kg/m^2^, compared to 39.3% in those with a BMI between 30 and 32 kg/m^2^. Low levels of prenatal activity were associated with an approximately 1.8-fold increase in the likelihood of operative delivery, consistent with findings demonstrating lower cesarean risk (relative risk 0.87) and shorter labor duration (62 minutes) associated with exercise [14]. In the Postpartum outcomes, the risk of postpartum hemorrhage was marginally elevated (atonic 5.4% vs. 8.3%; traumatic 3.6% versus 4.2%), for postpartum hemorrhage. In a similar study maternal obesity was found to exhibit a modest effect on postpartum hemorrhage risk which may differ by delivery mode [21]. Furthermore, they experienced longer hospital stays and a higher incidence of wound complications (29.2% infection, 25.0% dehiscence) [13] and initiated breastfeeding at slightly lower rates. Maternal obesity significantly exacerbates antepartum and intrapartum outcomes, while its effect on postpartum hemorrhage appears modest. Additionally, maternal obesity increases the risk of postpartum hemorrhage, particularly after vaginal deliveries (up to 19%), yet it may confer a protective effect against severe hemorrhage following cesarean sections [21]. The findings of our study are in accordance with a number of similar studies conducted globally in recent past [22, 23, 24, 25, 26, 27, 28, 29, 30, 31, 32-33]. The study by Vanlalfeli (2020), found maternal obesity to have a statistically significant correlation with several adverse outcomes, including hypertensive disorders, GDM, preeclampsia, cesarean section, induction of labor and postpartum complications and reported a positive association between maternal BMI and prolonged hospital stays, indicating a heavier healthcare burden [22]. Sunder *et al.* (2022) examined 2,972 pregnant women, of whom 1,657 had a BMI ≥30 and the findings revealed an increased risk of hypertension, antepartum hemorrhage and postpartum complications. Women with obesity were also more likely to experience hospital stays exceeding 5 days and found a reduced likelihood of intrauterine growth restriction [23]. The study by Lauth *et al.* (2021) on prolonged pregnancies, found that women with obesity, especially those with Class III obesity, experienced higher rates of induced labor and emergency cesarean sections and increased neonatal morbidity [24]. The study by Joewono *et al.* (2020), which involved 241 pregnant women with obesity, reported high rates of preeclampsia (25.8%), dyslipidemia (24.5%) and GDM (18.4%). Most deliveries (60.5%) were via cesarean section, with a significant number of neonates presenting low birth weight, asphyxia, or intrauterine fetal demise (IUFD). Notably, outcomes varied based on the site of antenatal care, indicating institutional disparities in managing maternal obesity [25]. Kutchi *et al.* (2020) applied the newer Asian Indian BMI threshold (≥25 kg/m^2^) and demonstrated significantly increased risks for a wide range of maternal complications, such as hypertensive disorders, preeclampsia, GDM and postpartum hemorrhage. Fetal complications included preterm birth, large for gestational age infants and increased NICU admissions, confirming that maternal obesity is a high-risk condition in Indian settings [26]. A more recent study by Wanaditya *et al.* (2023) found that obesity increased rates of hypertension (10.2-12.3%), preeclampsia (3-6.3%) and GDM (6.3-9.5%). Fetal risks included macrosomia (15.4-17.2%), preterm birth (4-5.5%) and structural anomalies such as neural tube defects. These complications often necessitated cesarean sections or labor inductions due to labor dystocia, fetal distress, or macrosomia [27]. In a review, Reed *et al.* (2023) from a perinatal perspective discussed how maternal obesity not only exacerbates comorbidities like insulin resistance and pregnancy-induced hypertension but also increases the risk of fetal anomalies, delivery complications, neonatal hypoxic-ischemic encephalopathy and long-term neurodevelopmental deficits in offspring [28]. Zehravi *et al.* (2021) found that obesity significantly increases maternal morbidity and the risk for conditions such as venous thromboembolism and postpartum hemorrhage, further elaborating on the link between obesity and gestational diabetes [29]. Castaneda *et al.* (2022) found a high prevalence of overweight or obesity (89.7%), with severe obesity in 17.9% by examining over 1,000 deliveries and inferred that obesity was associated with higher risks of macrosomia, neonatal hypoglycemia, NICU admissions and GDM whereas no association was found with COVID-19 infection severity [30]. A 2015 cross-sectional study found that compared to normal-weight women, obese women had higher odds of preeclampsia (OR = 3.67, 95% CI 2.57-5.24) and cesarean section (OR = 1.35, 95% CI 1.06-1.72), and their infants were more likely to be macrosomic (OR = 2.43, 95% CI 1.55-3.82). Indarti *et al.* (2021) found that while women with obesity had higher overall maternal and perinatal complications compared to the general population such as a high cesarean delivery rate (86.5%), increased gestational diabetes in obese I (50%), preeclampsia in obese II (34.4%) and preterm births in obese II (32.6%) [31]. Neal *et al.* (2022) reported that increasing obesity class is independently associated with a higher risk of adverse maternal and neonatal outcomes, including elevated rates of cesarean delivery, gestational diabetes and hypertensive disorders in women with obesity class III and above, as well as a significantly increased incidence of large-for-gestational-age (LGA) neonates, independent of maternal diabetes status [32]. Maternal obesity is associated with several adverse outcomes across pregnancy, labor, and the neonatal period. During pregnancy, it increases the likelihood of hypertensive disorders, including preeclampsia, as well as gestational diabetes. For the newborn, obesity in the mother is linked to a higher chance of macrosomia, greater need for specialized perinatal care, elevated risks of fetal mortality, abnormal fetal growth patterns, and congenital anomalies. Obesity also affects intrapartum outcomes, contributing to higher rates of labor induction, cesarean delivery, and postoperative wound complications [33]. Our single-center, cross-sectional study of 80 women with obesity limits causal inference and generalizability. Absence of a normal-BMI control group and reliance on self-reported or recorded pre-pregnancy weights may introduce bias. We did not comprehensively capture dietary, psychosocial, or structured exercise data and resource constraints precluded extensive endocrine and inflammatory profiling. Focusing on immediate peripartum and early postpartum outcomes also prevents assessment of long-term maternal and child health impacts.

## Conclusion:

Obesity during pregnancy presents significant risks at all stages of maternal and fetal care. In our cohort, younger women from middle and lower socioeconomic backgrounds showed a high prevalence of obesity. An elevated body mass index (BMI) was linked to higher rates of gestational diabetes, hypertensive disorders and anemia, with complication rates increasing alongside BMI. Intrapartum challenges, including prolonged and preterm labor, were more common and cesarean delivery rates significantly rose among women with a BMI of ≥ 35 kg/m^2^. Additionally, postpartum morbidity, particularly hemorrhage, wound infections and episiotomy dehiscence, was notably higher. Thus, we show the need for integrated strategies to mitigate the adverse effects of obesity on pregnancy and reduce its impact on obstetric services.

## Ethics of study:

Study was conducted after seeking approval from institutional ethics committee of Gajra Raja Medical College, Gwalior (M.P.)-India (108/IEC-GRMC/2022 dated 25/9/2023).

## Funding:

Nil

##  Authors' contributions:

Conception and design: SS, HR, MP

Analysis and interpretation of the data: SS, HR, AM

Drafting of the article: SS

Critical revision of the article for important intellectual content: HR, AM

Final approval of the article: SS, HR, MP, AM

Provision of study materials or patients: MP

Statistical expertise: AM

Administrative, technical, or logistic support: SS, MP

Collection and assembly of data: SS, HR
